# Spatial Memory Impairment Is Associated with Hippocampal Insulin Signals in Ovariectomized Rats

**DOI:** 10.1371/journal.pone.0104450

**Published:** 2014-08-06

**Authors:** Fang Wang, Yan-Feng Song, Jie Yin, Zi-Hua Liu, Xiao-Dan Mo, De-Gui Wang, Li-Ping Gao, Yu-Hong Jing

**Affiliations:** 1 Institute of Anatomy and Embryology, School of Basic Medical Sciences, Lanzhou University, Lanzhou, China; 2 Institute of Biochemistry and Molecular Biology, School of Basic Medical Sciences, Lanzhou University, Lanzhou, China; 3 Key Laboratory of Preclinical Study for New Drugs of Gansu Province, Lanzhou University, Lanzhou, China; 4 Maternal and Child Health Hospital of Gansu Province, Lanzhou, China; Hospital Infantil Universitario Niño Jesús, Ciberobn, Spain

## Abstract

Estrogen influences memory formation and insulin sensitivity. Meanwhile, glucose utilization directly affects learning and memory, which are modulated by insulin signals. Therefore, this study investigated whether or not the effect of estrogen on memory is associated with the regulatory effect of this hormone on glucose metabolism. The relative expression of estrogen receptor β (ERβ) and glucose transporter type 4 (GLUT4) in the hippocampus of rats were evaluated by western blot. Insulin level was assessed by ELISA and quantitative RT-PCR, and spatial memory was tested by the Morris water maze. Glucose utilization in the hippocampus was measured by 2-NBDG uptake analysis. Results showed that ovariectomy impaired the spatial memory of rats. These impairments are similar as the female rats treated with the ERβ antagonist tamoxifen (TAM). Estrogen blockade by ovariectomy or TAM treatment obviously decreased glucose utilization. This phenomenon was accompanied by decreased insulin level and GLUT4 expression in the hippocampus. The female rats were neutralized with hippocampal insulin with insulin antibody, which also impaired memory and local glucose consumption. These results indicated that estrogen blockade impaired the spatial memory of the female rats. The mechanisms by which estrogen blockade impaired memory partially contributed to the decline in hippocampal insulin signals, which diminished glucose consumption.

## Introduction

Estrogen deficiency following ovariectomy negatively affects learning and memory [1]. Decreased cognition is a defining characteristic of neurodegenerative diseases, including Alzheimer’s disease (AD) and Parkinson’s disease. Vearncombe and Pachana have reported that estrogen deficiency increases the risk of developing AD [2]. It is well known that the hippocampus is an essential region of learning and memory, synaptic plasticity provides a morphological basis for memory, and long-term potentiation (LTP) is a molecular biology foundation of memory. Consistently, in the hippocampus of rodents, learning behavior modification is usually accompanied by changes in synaptic plasticity factors, such as dendritic spine morphology and LTP. Animal studies have shown that ovariectomized (OVX) rats treated with estrogen replacement therapy (ERT) exhibit enhanced LTP and increased dendritic spine density in the CA1 to CA3 regions of the hippocampus [3]. Substantial evidence has proven the important effects of estrogen on learning and memory. However, the mechanisms by which estrogen affects memory formation remain unknown.

Glucose is the main source of energy in the brain. Uptaking of glucose is required by neurons during learning and memory. Alternatively, reduction of brain glucose metabolism caused the cognitive deficits. Therefore, normal glucose metabolism is crucial in improving and maintaining learning and memory. Glucose metabolism is regulated by a comprehensive molecular network. Among these molecules, insulin is an essential factor in this processing. Insulin-dependent glucose metabolism principally occurs in the hippocampus, and this process is mediated by glucose transporter type 4 (GLUT4) [4]. Previous study has been indicated that hippocampal neurons rapidly increase glucose utilization during hippocampal-dependent learning through the insulin-mediated translocation of GlLUT4 to the plasma membrane in rats [5]. Another study has been suggested that estrogen can increase insulin sensitivity and enhance insulin gene transcription and insulin release via estrogen receptors (ERs) [6]. Increasing literatures have been shown cross-talk occurred between estrogen and insulin signals during metabolism. Therefore, the present study aims to determine whether or not the effects of estrogen on learning and memory is associated with the insulin signals in OVX rats. Ovariectomy is a surgical procedure wherein the ovaries are removed, resulting in estrogen depletion. OVX rats are commonly used subjects in studies involving menopause and menopause-associated conditions. Results showed that the regulatory effect of estrogen on memory was dependent on ERβ. The effect of estrogen on memory formation partly contributed to the insulin signaling pathway in the hippocampus.

## Materials and Methods

### Animals

Adult female Sprague Dawley (SD) rats weighing 200 g to 250 g were purchased from the experimental center of Lanzhou University. The animals were maintained at 25±2°C and 12 h light-dark cycle. The animals were provided food and water ad libitum. All experimental protocols complied with the National Institutes of Health Guide for the Care and Use of Laboratory Animals and were approved by the Animal Ethics committee of Lanzhou University (permit number: SCXK Gan 2009-0004). All surgery was performed under chloral hydrate anesthesia, and all efforts were made to minimize suffering.

### Ovariectomy

Rats were intraperitoneally anesthetized with 7% chloral hydrate (360 mg/kg) and then subjected to ovariectomy. The ovaries were isolated by ligation of the most proximal vessel of the oviduct before removal. Sham rats were subjected to the same procedure without removing the ovaries. The animals recovered for 7 days after surgery.

### Cannulae implants and hippocampal injections

Rats were intraperitoneally anesthetized with 7% chloral hydrate (360 mg/kg). Then, stainless-steel guide cannulae were stereotactically implanted to bilaterally target the hippocampus according to the following coordinates: 3.6 mm posterior to the bregma, 2.00 mm lateral from the midline, and 3.5 mm ventral from the meninges. The rats were returned to their cages and allowed to recover from the surgery for 7 days. After training, the rats immediately received bilateral injections of drugs or saline. All hippocampal injections were performed in 2 µl per side. The injection needle was left in place for 10 min to allow complete absorption.

### Administration

The rats were divided into four groups: non-ovariectomy (normal), ovariectomy (OVX), non-ovariectomy + tamoxifen (N+TAM, 10 mg/kg, i.p.), and non-ovariectomy + anti-insulin antibody (N+anti, 2 µL/side, bilateral hippocampal injection, Santa Cruz, Dallas, USA). TAM (Yangzi Pharmacology, Jiangsu, China) was dissolved into normal saline by sonication to become injectable suspension with a final concentration of 5 mg/mL. After behavioral training, rats in the N+TAM group were immediately intraperitoneally injected with TAM (10 mg/kg, once a day), rats in the N+anti group were immediately injected with anti-insulin antibody into bilateral hippocampous (2 µl per hippocampus, once a day), rats in other groups were injected with saline as control for five consecutive days.

### Spatial memory assay by the Morris water maze

A circular pool (diameter: 120 cm; depth: 50 cm) was filled with 23°C water to a height of 2 cm above a platform (10 cm×10 cm). The pool was divided into four quadrants (i.e. quadrants 1 to 4). Four quadrants were labelled with different shape and color paper on the wall of circular pool. The platform was placed in a constant location in the middle ring of quadrant 3. The rats were trained for 5 days with the hidden platform. Each trial was started by placing a rat with its back facing toward the platform at the starting points. The trial was terminated when the rat stood on the platform. However, when the rat did not find the platform within 60 s, it was guided on the platform for 15 s. During acquisition (days 1 to 5) of the spatial navigation test, all groups were given one session of four trials each day. Each day after training, the rat was removed from the pool, dried, and then returned to its cage. In spatial probe test, the platform was removed, and the rats were allowed to swim for 60 s on day 6. The number of times the rat crossed over and the time spent in the goal quadrant were recorded and analyzed. The data obtained from the Morris water maze task were automatically recorded through a WMT-100 analysis system (Taimeng, Chengdu, China).

### Glucose uptake evaluation

2-NBD glucose (2-NBDG, Invitrogen, California, USA) was dissolved in normal saline with a final concentration of 2 mmol/L. Rats were injected with 2-NBDG into bilateral hippocampus (2 µl per hippocampus) at six hours before the special probe test. After the probe test, the rats were subjected to cardiac perfusion with normal saline followed by 4% paraformaldehyde. The brains of the rats were removed, postfixed in 4% paraformaldehyde overnight, and then dehydrated sequentially in 20% and 30% sucrose solutions in 0.1 M phosphate buffer (pH 7.4) until they sank. Frozen sections throughout the hippocampus were cut using a freezing microtome (Leica, Germany) at a thickness of 30 µm and stored at −20°C in a cryoprotectant solution. Six sections were selected from each rat according to the atlas of rats brain, i.e., at −3.30, −3.45, −3.60, −3.75, −3.90, and −4.05 mm from the bregma. The number of 2-NBDG-positive cells in hippocampal CA3 area was counted per section under a fluorescence microscope to assess the rate of glucose uptake.

### Insulin level in hippocampus by ELISA

The hippocampus was dissected and homogenized with lysis buffer (10 mM Tris-HCl, pH 7.4, 150 mM NaCl, 0.8 M EDTA, 0.5 M EGTA, 1% Triton X-100, 0.5% Nonidet P-40, and 1% protease cocktails). The lysates were then centrifuged at 12,000 *g* for 30 min, and the supernatant was collected. The samples were diluted, added the standards and samples to the coated microplate, and then incubated at 37°C for 30 min. After the liquid in each well was discarded, the wells were dried and then rinsed with washing buffer. Each well was added with a horseradish peroxidase (HRP)-conjugated reagent, incubated at 37°C for 30 min, and then washed. Finally, each well was added with a chromogen solution and then was incubated at 37°C for 15 min in the dark. The absorbance was measured at 450 nm after adding a stop solution within 15 min.

### Western blot analysis

After behavioral tests, the rats were immediately sacrificed by decapitation. The brains of the rats were immediately removed and then placed on ice-cold plates. The hippocampus was dissected, frozen in liquid nitrogen. Tissues of hippocampus were homogenized with RIPA buffer (50 mM Tris PH 7.4, 150 mM NaCl, 1% Triton X-100, 1% sodium dexycholate, 0.1% SDS and protease inhibitors.) and the total ptotein was extracted, then preserved at −80°C for subsequent western blot analysis. Proteins were fractionated through sodium dodecyl sulfate–polyacrylamide gel electrophoresis and then transferred onto polyvinylidene fluoride membranes. The membranes were blocked with 5% nonfat powdered milk (Shenggong, Shanghai, China) in TBST buffer at room temperature for 2 hours. The membranes containing fractionated proteins were incubated overnight in 1∶500 dilution of anti-GLUT4 (Santa Cruz, Dallas, USA) and anti-ERβ (Santa Cruz, Dallas, USA) at 4°C, respectively. The membranes were incubated with an HRP-conjugated secondary antibody (ZSGB-BIO, Beijing, China, 1: 5000) at room temperature for 60 min. GAPDH (Millipore, Billerica, USA, 1∶5000) was used as a loading control. Densitometry analysis was performed using Image J software.

### Quantitative real-time RT-PCR

After brain removal, the tissue samples were rinsed with ice-cold saline and then placed on the ice-plat. The hippocampus was dissected and then frozen in liquid nitrogen. Total RNA in the hippocampus was isolated using an RNA isolation kit from Takara. RNA quantity was measured by spectrophotometrical quantification (NanoDrop 2000, Peqlab, Erlangen, Germany). Total RNA (1.0 µg) was reverse transcribed to cDNA. Quantitative real-time PCR was performed with Quanti-Fast SYBR Green PCR Master mix (BIO-RAD, Hercules, USA) using Rotorgene 3000 system (Corbett, Sydney, Australia). The primers for rat insulin were as follows: forward, 5′-CAG CAC CTT TGT GGT TCT CAC TT-3′ and reverse, 3′-CTC CAC CCA GCT CCA GTT GT-5′. GAPDH was used as internal control. The primers used were as follows: forward, 5′-GCC ACA GTC AAG GCT GAG AAT G-3′ and reverse, 3′-ATG GTG GTG AAG ACG CCA GTA-5′. The PCR conditions were as follows: 95°C for 10 s, followed by 40 cycles of 95°C for 5 s and 60°C for 30 s. Gene expression was normalized to the expression of the reference gene GAPDH.

### Statistical analysis

All data were expressed as mean±SEM. Firstly, homogeneities of variance were evaluated, then multiple comparisons were performed using one-way ANOVA, two groups comparisons were analyzed by unpaired student’s test. Statistical significance was considered at P<0.05.

## Results

### Ovariectomy decreased ERβ expression in the hippocampus

**Figure 1:**
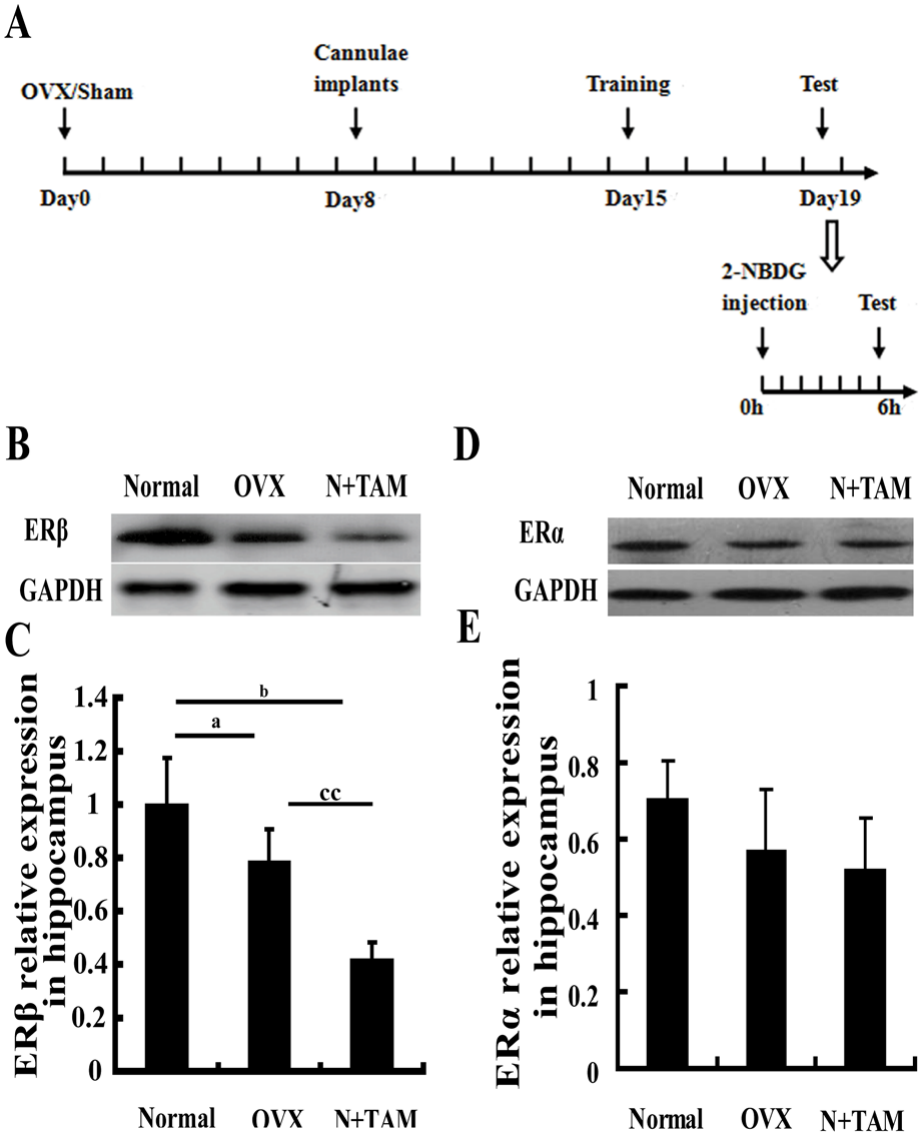
Estrogen deficiency declined ERβ expression in the hippocampus. (A) Schematic graph of time course. (B) Representative immunoblots showing the protein levels of ERβ in the hippocampus for different experimental conditions. (C) ERβ expression was quantified through graph depicting the densitometric analysis of scanned western blots. (D) Representative immunoblots showing the protein levels of ERα in the hippocampus for different experimental conditions. (E) ERα expression was quantified through graph depicting the densitometric analysis of scanned western blots. ^a^P<0.05, OVX group vs. normal group. ^b^P<0.05, N+TAM group vs. normal group. ^cc^P<0.01, N+TAM group vs. OVX group. *n* = 6.

The effect of estrogen on memory is mainly regulated through ERβ [7]. Thus, we first observed ERβ expression in the hippocampus by western blot. The expression level of ERβ in the hippocampus significantly decreased in the OVX rats compared to the normal rats (Figures 1B and 1C). TAM treatment significantly diminished the expression of ERβ compared with non-ovariectomy and OVX groups (Figures 1B and 1C). ERα expression in hippocampus was also evaluated using western blotting, mild decreased in OVX and TAM groups compared to the normal group, but no significantly difference among three groups (Figures 1D and 1E).

### Effect of estrogen deficiency on spatial memory

**Figure 2:**
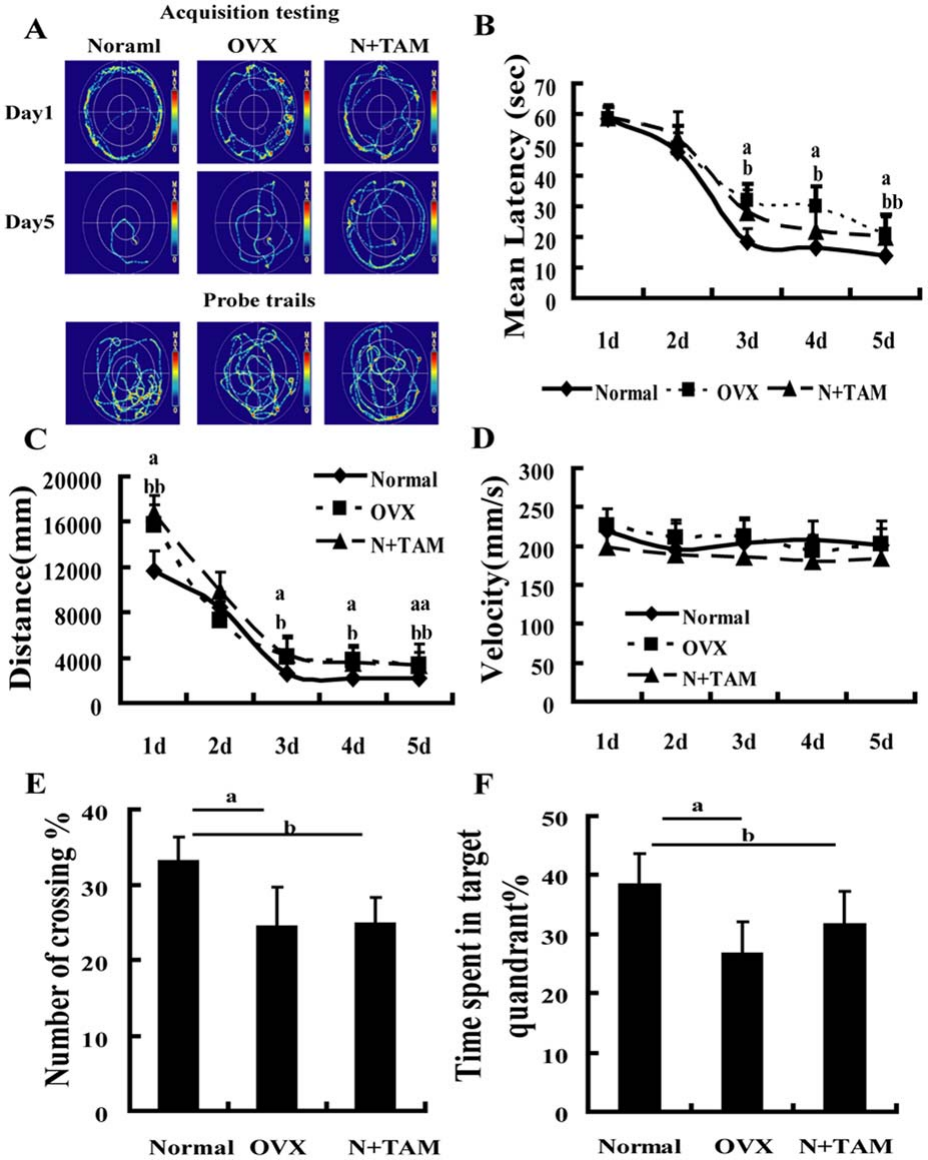
Estrogen deficiency induced spatial memory impairment. (A) Representative traces by the Morris water maze. The performance of rats in the Morris water maze using hidden platform training for 5 days was assessed. (B) Escape latency (One-way ANOVA of the escape latency revealed significant differences in the OVX group compared to the normal group at day 3 to 5, F_1,24_ = 5.053, P = 0.041; F_1,24_ = 8.093, P = 0.013 and F_1,24_ = 5.473, P = 0.035, respectively. Signficant difference in the N+TAM group vs. the normal group at day 3 to 5, F_1,28_ = 7.259, P = 0.015; F_1,28_ = 4.801, P = 0.046; F_1,28_ = 8.771, P = 0.008. No significant differences between the N+TAM group and the OVX group). (C) Distance to the platform (One-way ANOVA of the distance to the platform showed significant differences in the OVX group and N+TAM group at day 3 to 5 compared to the normal group. OVX group vs. normal group at day 3 to 5, F_1,24_ = 4.633, P = 0.043; F_1,24_ = 5.830, P = 0.023; F_1,24_ = 12.851, P = 0.001, respectively. N+TAM group vs. normal group at day 3 to 5, F_1,28_ = 5.034, P = 0.031; F_1,28_ = 4.939, P = 0.038; F_1,28_ = 8.599, P = 0.006, respectively. No significant differences between the N+TAM group and the OVX group). (D) Swimming velocity in the normal, OVX, and N+TAM groups (One-way ANOVA of the swimming velocity showed no significant difference in the OVX group and N+TAM group compared to the normal group at day 1 to 5). (E) Percentage of number of crossing and (F) time spent in the target quadrant during the probe trial. ^a^P<0.05, OVX group vs. normal group. ^b^P<0.05 and ^bb^P<0.01, N+TAM group vs. normal group, n = 8.

The spatial memory was evaluated by the Morris water maze after ovariectomy or TAM treatment. The average escape latencies and distances at day 3 to 5 of training were longer in the OVX rats and TAM-treated rats than in the normal rats (Figures 2A to 2C). The probe trial test showed that the percentage of crossing and time in the target quadrant decreased in the OVX and N+TAM rats than in the normal rats (Figures 2E and 2F). The swimming velocity showed no significant difference among the three groups (Figure 2D). These results indicated that OVX or TAM treatment impaired the spatial memory of rats.

### Effect of estrogen deficiency on glucose utilization in the hippocampus

**Figure 3:**
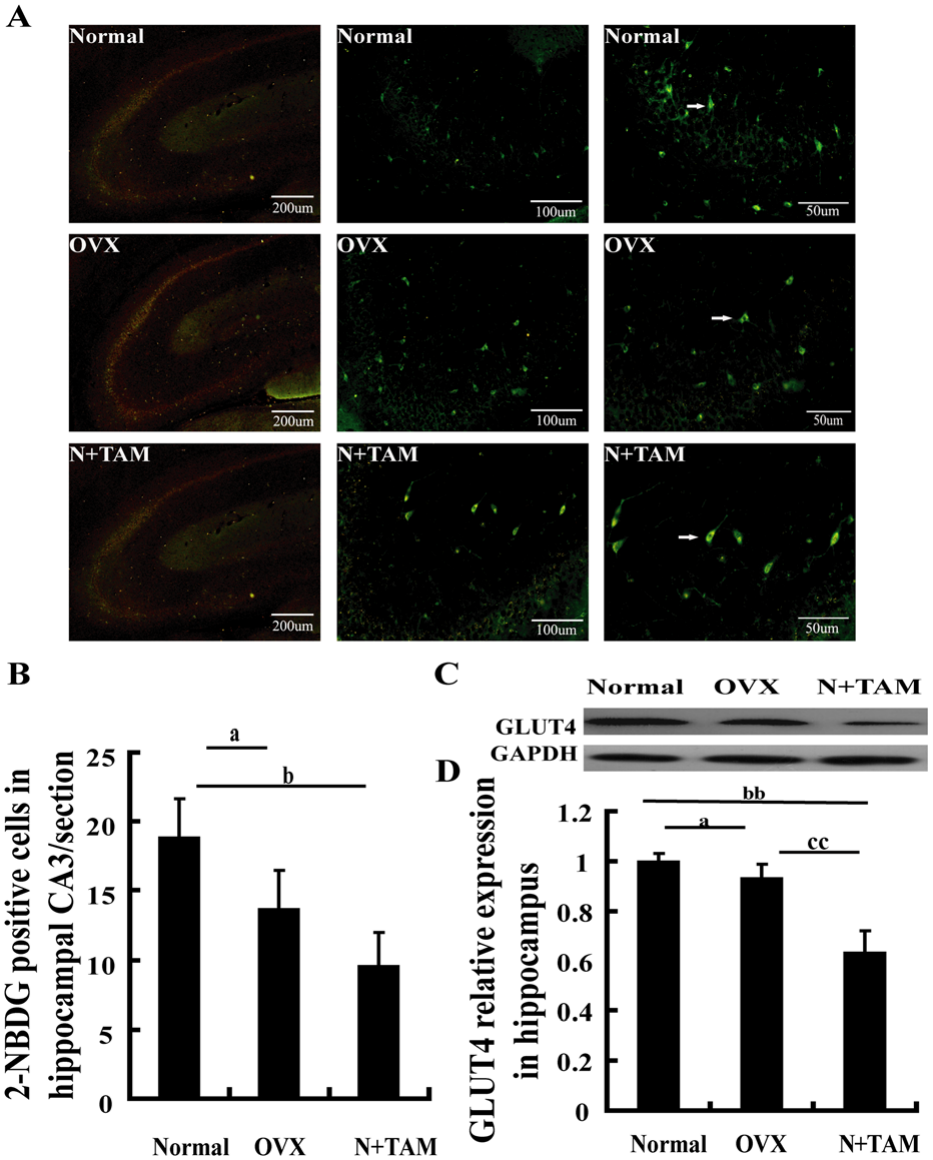
Estrogen deficiency inhibited glucose uptake in the hippocampus. (A) Representative images showing 2-NBDG positive cells at CA3, white arrows indicate the 2-NBDG positive cells. (B) 2-NBDG-positive cells at CA3 were fewer in the OVX and N+TAM rats than in the normal rats. (C) Representative immunoblots showing the protein levels of GLUT4 in the hippocampus under different experimental conditions. (D) Graph depicting the densitometric analysis of scanned western blots. ^a^P<0.05, OVX group vs. normal group.^ b^P<0.05 and ^bb^P<0.01, N+TAM group vs. normal group. ^cc^P<0.01, N+TAM group vs. OVX group. *n* = 8.

Glucose-uptaking is required by neurons during learning and memory [8]. Positron emission tomography has shown that patients with cognitive dysfunction have an abnormal glucose metabolism rate in the brain and that the degree of impairment in glucose metabolism is related to dementia severity [9]. Accordingly, we observed whether or not ovarian hormone loss alters glucose uptake in hippocampal CA3 area. As shown in Figures 3A and 3B, the number of 2-NBDG-positive cells in hippocampal CA3 area was lower in the OVX and N+TAM rats than in the normal rats. OVX or TAM also decreased the expression level of GLUT4 in the hippocampus. Furthermore, the lower expression of GLUT4 in TAM group than that in the OVX group (Figure 3C, D).

### Effect of estrogen deficiency on insulin production

**Figure 4:**
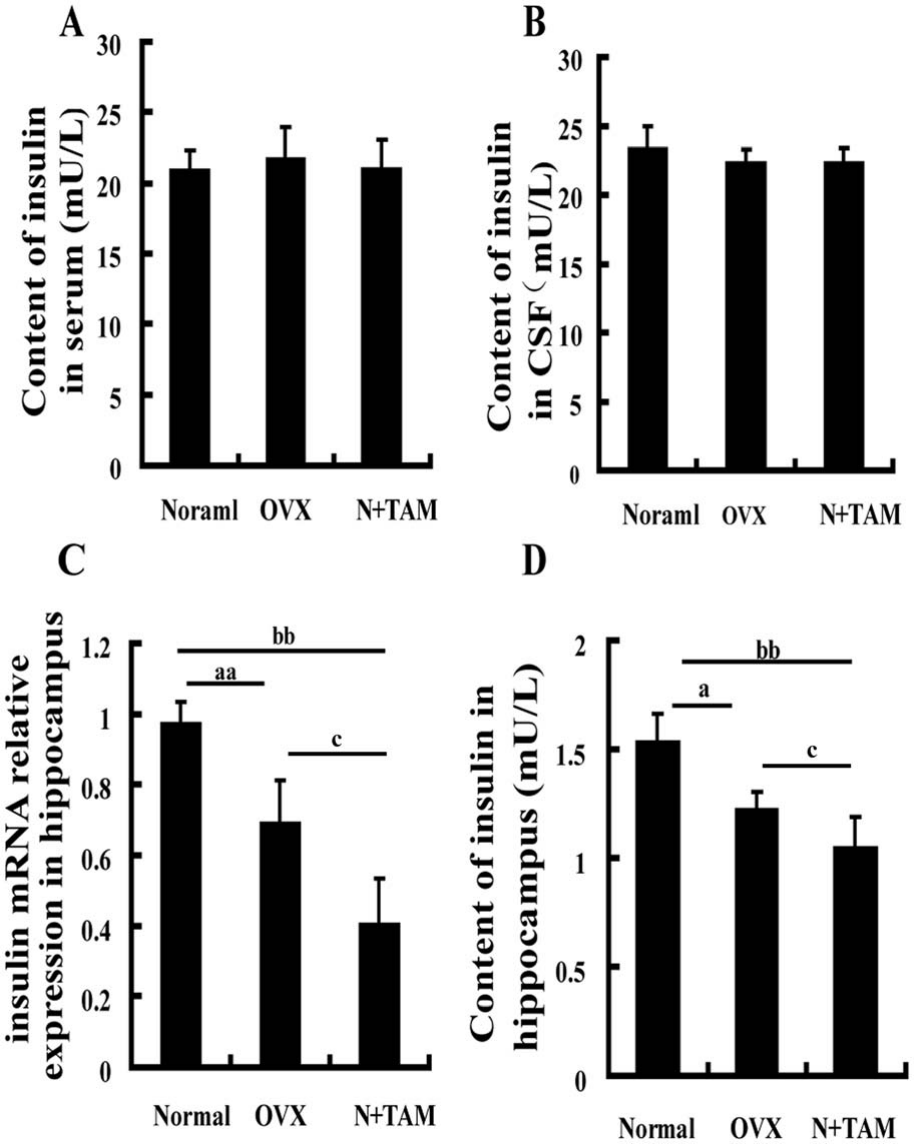
Low estrogen levels decreased insulin expression in the hippocampus. Insulin content in the serum (A) and CSF (B). (C) Insulin mRNA expression in the hippocampus was measured by quantitative RT-PCR. (D) Evaluation of insulin content in the hippocampus using ELISA. ^a^P<0.05 and ^aa^P<0.01, OVX group vs normal group. ^b^P<0.05 and ^bb^P<0.01, N+TAM group vs. normal group. ^c^P<0.01, N+TAM group vs. OVX group. *n* = 8.

Given the changes in glucose utilization and GLUT4 expression in the hippocampus following estrogen deficiency, we determined whether or not estrogen deficiency influences insulin production. No significant differences in insulin contents in the serum and cerebrospinal fluid were observed among the three groups (Figures 4A and 4B). However, the mRNA level of insulin was reduced by 30% and 50% in the hippocampus of the OVX and N+TAM rats compared with the normal rats (Figure 4C). At the protein level, the insulin content in the hippocampus was 1.22 mU/L in the OVX rats, 1.04 mU/L in the TAM rats, and 1.53 mU/L in the normal rats, as evaluated using ELISA, and insulin levels of both mRNA and protein are lower in TAM group compared to the normal and OVX groups (Figure 4D).

### Effect of hippocampal insulin blockade on local glucose utilization

**Figure 5:**
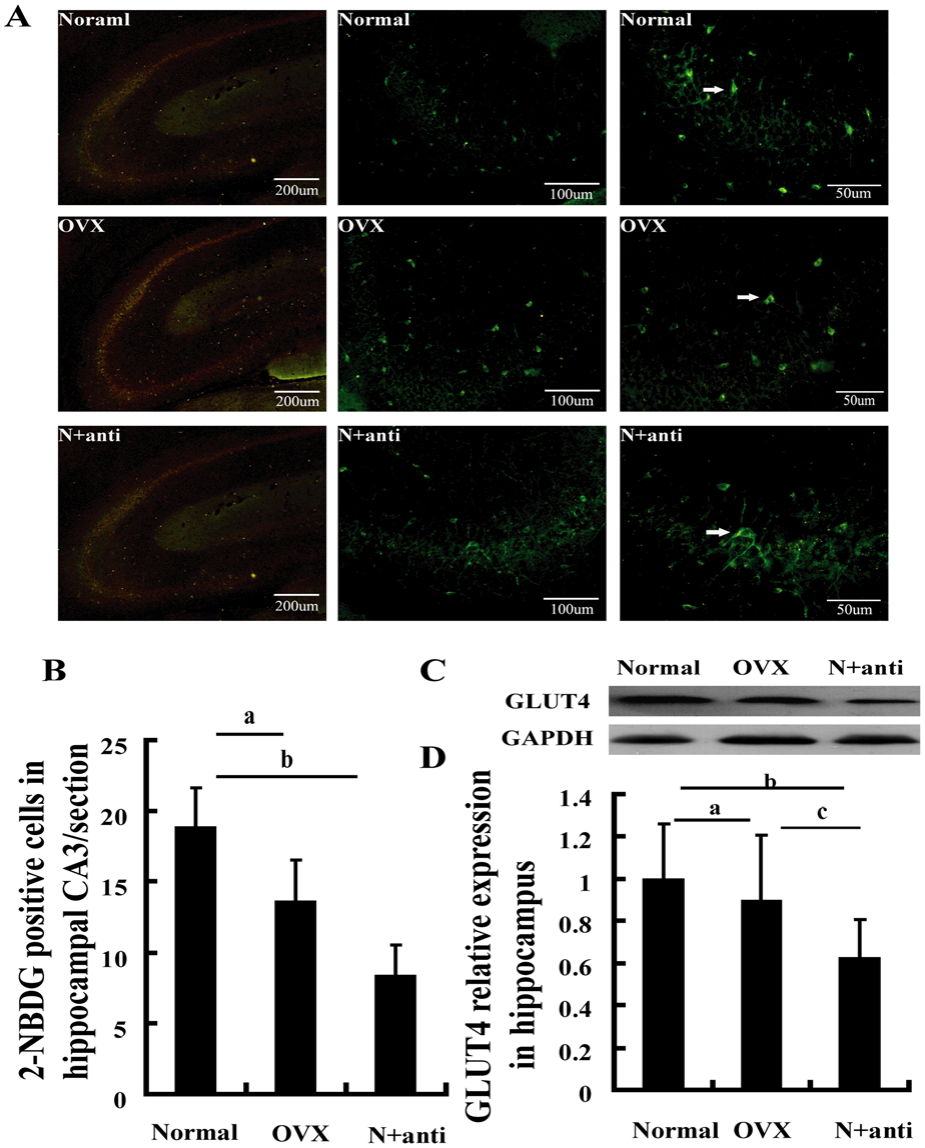
Insulin deficiency inhibited glucose uptake in the hippocampus. (A) Representative images showing 2-NBDG positive cells at CA3, white arrows indicate the 2-NBDG positive cells. (B) 2-NBDG-positive cells at CA3 were fewer in the OVX and N+anti rats than in the normal rats. (C) Hippocampal tissues were used for western blot with GLUT4 and GAPDH antibodies. (D) Densitometric analysis of scanned western blots. ^a^P<0.05, OVX group vs. normal group. ^b^P<0.05, N+anti group vs. normal group. ^c^P<0.05 and ^cc^P<0.01, N+anti group vs. OVX group. *n* = 8.

We assessed whether or not hippocampal insulin blockade directly decreases glucose utilization and downregulates GlUT4 expression by neutralization with insulin antibody. The number of 2-NBDG-positive cells in hippocampal CA3 area was reduced by 50% in the N+anti rats compared with that in the normal rats (Figures 5A and 5B). Hippocampal GLUT4 expression also decreased in the N+anti rats compared with the normal rats (Figure 5C, D).

### Effect of hippocampal insulin blockade on spatial memory

**Figure 6:**
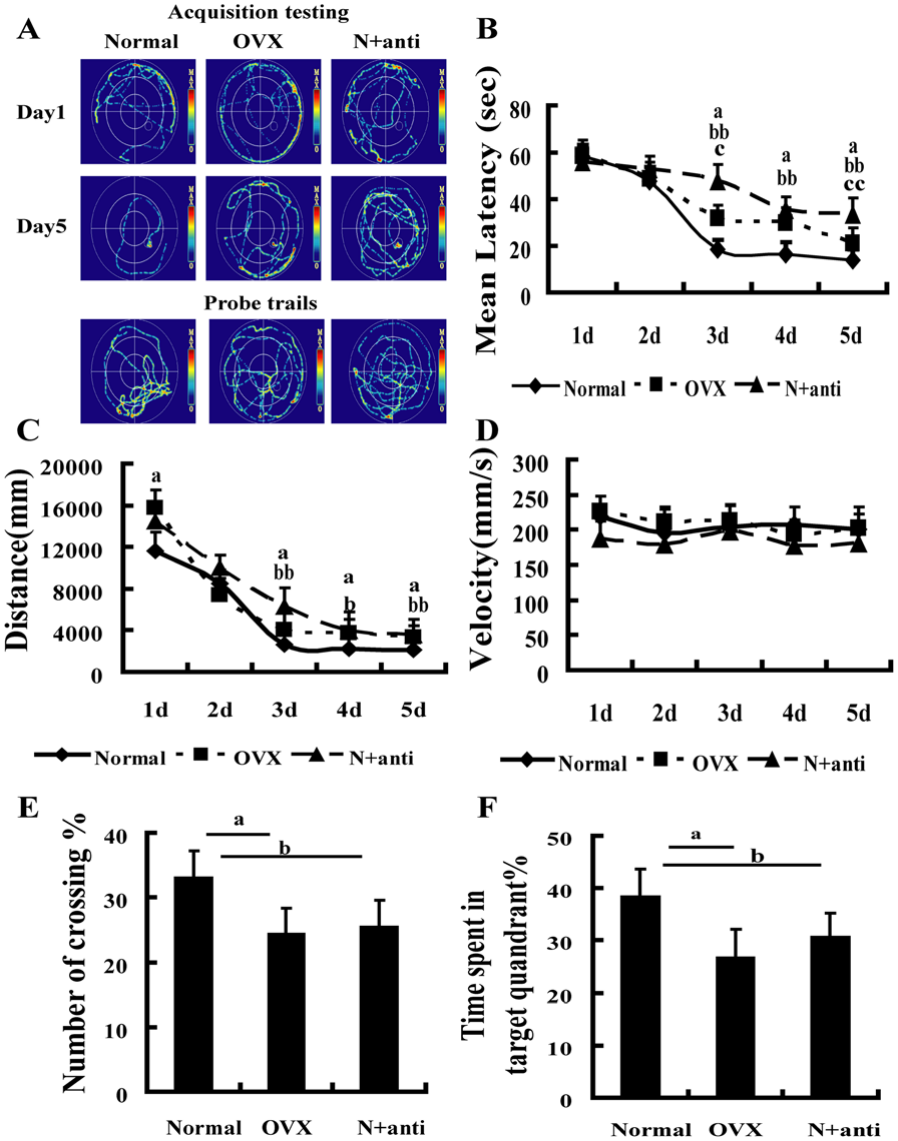
Neutralized hippocampal insulin by insulin antibody induced memory deficiency. (A) Representative traces of the Morris water maze. The performance of rats in the Morris water maze using hidden platform training for 5 days was assessed. (B) Escape latency (One-way ANOVA of the escape lantency showed significant differences in the OVX group and N+TAM group at day 3 to 5 compared to the normal group. OVX group vs. normal group at day 3 to 5, F_1,24_ = 4.633, P = 0.043; F_1,24_ = 5.830, P = 0.023; F_1,24_ = 12.851, P = 0.001, respectively. N+anti group vs. normal group at day 3 to 5, F_1,29_ = 30.190, P = 4.9×10^−5^; F_1,29_ = 23.625, P = 1.2×10^−4^; F_1,29_ = 49.614, P = 1.4×10^−6^, respectively. No significant differences between N+anti group and OVX group at day 3 to 5). (C) Distance to the platform (One-way ANOVA of the distance to the platform showed significant differences in the OVX group and N+TAM group at day 3 to 5 compared to the normal group. OVX group vs. normal group at day 3 to 5, F_1,24_ = 4.633, P = 0.043; F_1,24_ = 5.830, P = 0.023; F_1,24_ = 12.851, P = 0.001, respectively. N+anti group vs. normal group at day 3 to 5, F_1,29_ = 12.321, P = 0.002; F_1,29_ = 5.134, P = 0.029; F_1,29_ = 17.437, P = 3.4×10^−4^, respectively. No significant differences between N+anti group and OVX group). (D) Swimming velocity in the normal, OVX, and N+anti groups (One-way ANOVA of the swimming velocity in the OVX group and N+TAM group showed no significant differences compared to the normal group at day 1 to 5). (E) Percentage of number of crossing and (F) time spent in the target quadrant during the probe trial. ^a^P<0.05, OVX group vs. normal group. ^b^P<0.05 and ^bb^P<0.01, N+anti group vs. normal group. ^c^P<0.05 and ^cc^P<0.01, N+anti group vs. OVX group. *n* = 8.

Spatial memory was evaluated by the Morris water maze. The average escape latencies and distances at day 3 to 5 of training were longer in the OVX and N+anti rats than in the normal rats (Figures 6B and 6C). The percentage of crossing and the time in the target quadrant were obviously reduced in the N+anti and OVX rats compared with the normal rats during the probe trial (Figures 6E and 6F). The swimming velocity showed no difference among the three groups (Figure 6D).

## Discussion

The present study found that estrogen blockade by ovariectomy or TAM treatment impaired the spatial memory of rats. This finding is consistent with clinical observation. Women who undergo surgical menopause without hormone treatment have increased risk for cognitive impairment and dementia in their later life [8]. Ovariectomy or TAM treatment decreased the expression of ERβ in the hippocampus; this phenomenon was accompanied by reduced glucose utilization in the hippocampus. Different behavioral paradigms have related glucose uptake to learning and memory in discrete brain regions [9]. In the hippocampus, insulin-dependent glucose metabolism mainly occurs and is mediated by GLUT4 [4]. Therefore, we studied whether or not the content of insulin in the hippocampus was changed after ovariectomy or TAM treatment. The levels of insulin and the expression of GLUT4 in the hippocampus were lower in the OVX and N+TAM rats than in the normal rats. The hippocampal insulin neutralized with insulin antibody also impaired memory and local glucose consumption. These results indicated that estrogen blockade impaired the spatial memory of the rats via ERβ. This effect partially contributed to the decline in hippocampal insulin, which diminished glucose consumption. Emerging studies have associated ovarian hormone loss with a marked decrease in synaptic strength at CA3; the synaptic strength at CA3 to CA1 has been associated with hippocampus-dependent memory [10]. The activity-dependent glucose uptake in discrete brain regions is associated with a specific behavior, which is controlled by corresponding brain regions [4]. In the present experiment, the glucose uptake at CA3 of hippocampus was observed by 2-NBDG injection. The results indicated that spatial memory impairment was accompanied by reduced number of 2-NBDG-positive cells at CA3 of hippocampus.

Estrogen replacement therapy (ERT) prevents cognitive decline in the postmenopausal women [2]. However, ERT with high levels of estradiol has a several side-effects including the risk of cancer suffering. The effect of exogenous estradiol administration on OVX female rats depends on the dose [11]. For example, therapy with low dose of estradiol improves the performance of OVX rats in Morris water maze tasks [12], whereas therapy with high levels of estradiol disrupts the performance of these rats [13]. Postmenopausal women are prone to develop gynecological diseases, such as breast cancer and uterine myoma, which are related to high sensitivity to estrogen [14], [15]. ER antagonists are used to treat the breast cancer, for example TAM [16]. However, our present experiment suggested that the ER antagonist TAM may have some negative effects, especially on spacial learning and memory**.** Consistently, Jaime and his collegues’s study has been shown that women using TAM for the treatment of early breast cancer, scored significantly worse than controls on tasks of immediate and delayed visual memory, verbal fluency, immediate verbal memory, visuo-spatial ability, and processing speed, suggesting TAM use in pre-menopausal breast cancer may be associated with cognitive difficulties [17]. Similarly, another research team has reported that women with breast cancer taking TAM showed significantly lower semantic memory scores, smaller hippocampal volumes and lower glucose utilization in the cerebral region compared with the control grooup [18]. Alternatively, Paul Newhouse and his colleagues have reported that TAM significantly attenuated the impairment from cholinergic blockade on tasks of verbal episodic memory and spatial navigation, but effects on attentional/psychomotor tasks were more variable compared with placebo treatment. Further analysis by APOE genotype showed that APO ε4^+^ women showed a greater beneficial effect of TAM on reversing the cholinergic impairment than APO ε4^−^ women on most tasks [19]. These discrepancies may be due to the subject’s conditions, different therapeutic protocol and different behavioral paradigm.

In the present study, we found that the insulin content in the hippocampus was decreased in the OVX and N+TAM rats. Glucose utilization was also decreased in the hippocampus after estrogen blockade. In the past decade, studies have indicated that estrogen binds to its receptor, translocates to the nucleus, targets a specific gene sequence, and then regulates gene activity [20]. The gene motif of estrogen target has been found in the promoter of insulin, suggesting that estrogen can directly regulate insulin expression [21]. Estrogen can also enhance insulin sensitivity by phosphorylating insulin receptors [22]. These findings suggested that estrogen can regulate insulin production and signals. Some studies have indicated that the regulatory effect of estrogen on synaptic plasticity is mediated by ERβ [22]. Synaptic plasticity is one of the critical foundations in learning and memory [23].

Estrogen is mainly generated from ovaries. Emerging studies have suggested that neurons can generate a minimal amount of estrogen [24], which may facilitate transient regulation of local brain metabolism [25]. However, extensive studies must be performed to elucidate the functions of brain-derived estrogen. Both ERβ and ERα have been observed in most brain regions [20]. However, the functions of different receptors in learning and memory remain controversial [26]. In present study, we found estrogen deficiency impaired the learning and memory mainly mediated by ERβ in rats. The distribution of ERβ in hippocampus of rat were modulated by the fluctuation of estrogen during oestrus cycle, but the ERα is a little changes in hippocampus during oestrus cycle, suggesting the ERβ is more sensitive to estrogen fluctuation than the ERα in hippocampus [27]. Further studyes have been suggested that ERβ levels are greater than ERα in human and rat hippocampus [28]. But contradiction with our results, Na Qu et al. showed that the expression of ERα, but not ERβ, was decreased in the hippocampus starting 1 wk after ovariectomy [29]. It is very difficult to interpreted this contradiction, may be due to ERα is more likely to be localized to the nucleus [30], [31], at early stage of ovariectomy, ERα was implicated.

Although the mechanisms by which estrogen influences learning and memory are complicated and obscure, the present results suggested that estrogen deficiency can impair learning and memory mediated by ERβ. These effects were associated with reduced insulin and glucose utilization.
